# Competence, preparation, and relaxation: Contributing factors to EFL student teachers' self-efficacy and teaching performance in microteaching training

**DOI:** 10.1016/j.heliyon.2024.e26216

**Published:** 2024-02-17

**Authors:** Zhanni Luo, Huazhen Li

**Affiliations:** School of Foreign Languages and Literatures, Chongqing Normal University, China

**Keywords:** Microteaching, Teacher training, EFL teacher, Self-efficacy, Teaching performance, Structural equation modeling (SEM)

## Abstract

Microteaching is called “micro” teaching because it involves teaching a short lesson to a small group of people in a simulated classroom setting, with the goal of improving specific teaching skills or behaviors. Microteaching training represents a significant approach for enhancing the teaching competencies of student teachers. However, there is a scarcity of studies that examine the factors contributing to the self-efficacy and teaching performance of student teachers, both of which are central concerns in microteaching training programs. This study addresses this gap by synthesizing five contributing factors from existing literature, collecting survey responses from 272 English-as-a-foreign-language (EFL) student teachers, and employing structural equation modeling (SEM) to analyze the relationships between these factors. The four hypotheses that were rejected yielded unexpected results, indicating negative relationships between participants' teaching experience and EFL speaking competence with their lesson-delivery competence, as well as a negative relationship between lesson-delivery competence and self-efficacy. Interestingly, public speaking anxiety was found to have no statistically significant impact on EFL student teachers' self-efficacy. This study establishes a theoretical framework that can assist decision-makers in enhancing facilitators and overcoming barriers in microteaching training programs. This framework can also be adapted for use in other academic studies.

## 1 Introduction

### 1.1 Definition and background

Microteaching is an effective approach for developing teaching competencies in pre-service and in-service teachers, and it is widely used in teacher education programs worldwide [1,2]. Microteaching is called “micro” teaching because it involves teaching a short lesson to a small group of people in a simulated classroom setting, with the goal of improving specific teaching skills or behaviors [3].

Microteaching training is cyclical and involves a four-step process: plan, teach, observe, and critique [4]. Teachers make teaching plans, give classes, invite peers to observe, and receive critiques on their teaching performance from peers, supervisors, or teacher educators. Then, teachers are expected to refine their teaching plans based on the critiques, remake the teaching plan, and teach again [5,6]. This approach helps teachers reflect on their teaching strategies and identify areas for improvement [7].

Microteaching training is based on microteaching systems, which are technological tools designed to facilitate the implementation and management of microteaching in teacher education. These systems consist of micro-classrooms, a central control room, and a suite of microteaching management software. In addition to the essential components found in a typical classroom, such as a blackboard, digital screen, computer, teaching platform, and tables/chairs for students, a micro-classroom features a video recording and transmission system that sends footage to the central control room. Through the microteaching management software in the central control room, the teacher instructor can simultaneously oversee multiple micro-classrooms, monitor teachers' progress, and provide timely feedback using microphones connected to each micro-classroom. Student teachers can also use the video recording system equipped in the micro-classroom to record their simulated teaching sessions. They can review and analyze these recordings after class to pinpoint teaching problems, address them, and ultimately improve teaching skills.

Overall, the microteaching system provides a safe and controlled environment for student teachers or pre-service teachers to practice their teaching skills. This enables them to enhance the effectiveness of their instruction before stepping into actual classroom scenarios, as supported by Refs. [2,8,9]. Microteaching plays an important role in teacher training.

### 1.2 Research gaps

Researchers are keen on identifying the factors contributing to the enhancement of teaching performance in microteaching training sessions among student teachers or pre-service teachers. Prior studies have identified several factors, such as motivation [10], emotional intelligence [11], teaching anxiety [12], and self-efficacy [13,14].

Nonetheless, previous research in the field of teacher education has primarily concentrated on affective factors like motivation and anxiety, while giving limited attention to non-affective factors. This research gap is especially noticeable for student teachers, whose professional development is influenced by various unstable non-affective factors, including teaching experience, language proficiency, and lesson-delivery competence.

Moreover, there is a noticeable dearth of research that specifically examines English-*as*-a-foreign-language (EFL) teachers. Speaking a second language itself can pose a significant challenge [15], yet very few studies have explored the impact of EFL teachers' language proficiency or their level of public speaking anxiety on their teaching effectiveness. Consequently, there is an urgent need for further investigation into the relationship between these factors and EFL teachers' teaching performance.

### 1.3 Research aims and significance

Given the frequent mention of self-efficacy in prior studies, we have drawn a parallel between it and teaching performance. Consequently, the primary aim of this study is to investigate the factors that exert influence on the self-efficacy and teaching performance of EFL student teachers within microteaching training programs.

We conducted a comprehensive analysis of the factors impacting teacher self-efficacy and teaching performance as documented in pertinent literature. These factors were meticulously categorized, culminating in the identification of five key elements: teaching experience (TE), EFL speaking competence (ESC), lesson-delivery competence (LDC), lesson preparedness (LP), and public-speaking anxiety (PSA). Subsequently, we employed a structural equation model to scrutinize this framework. Ultimately, this study seeks to answer the question “What are the interrelationships among the seven factors within the theoretical framework in microteaching training programs?”

The ensuing findings will shed light on the critical factors in microteaching training, offering valuable insights for future decision-making in educational interventions. Furthermore, the theoretical framework synthesized in this study can be readily applied or adapted by other researchers in related studies.

## 2 Theoretical framework and hypotheses

### 2.1 Theoretical framework

To develop their teaching abilities, teachers must possess fundamental knowledge and skills, which encompass a range of teaching competencies, including pedagogical content knowledge [16], prior knowledge of teaching [17], communicative skills, and professional and common cultural knowledge [18]. We further divided the “fundamental knowledge and skills” category into two parts: teaching knowledge and EFL speaking competence.

Teaching knowledge has been studied previously under different names, such as foundational knowledge [19], professional knowledge [20], pedagogical content knowledge [16], prior knowledge of teaching [17], practical knowledge [17], working knowledge [21], content knowledge [22], and professional competence [23].

EFL speaking competence was added specifically for the research objectives of this study, which focuses on EFL student teachers. As explained in the introduction section, few previous studies have examined foreign language teachers, despite the challenges of speaking another language. We believe that for an EFL teacher, both linguistic and pedagogical competencies are essential. The importance of EFL speaking competence has also been emphasized in studies of Daud, Ras, Novitri and Audia [24], Teksan, Mutlu and Cinpolat [25], and Ozenc, Orhan-Karsak and Ozenc [26].

In the pre-teaching preparation stage, one key factor is lesson preparedness, which refers to the level of ability or readiness to complete necessary tasks in delivering courses [27]. Previous studies have presented seemingly distinct but highly related terms such as lesson plans [28], course design [27], instructional planning [26], and planning and preparation [16].

After reviewing the literature on lesson preparedness, we identified another crucial factor: lesson-delivery competence. This encompasses several aspects, including in-class time management [27] and discipline management [29].

Lesson-delivery competence also involves managing the teaching process, such as achieving predetermined teaching goals, providing appropriate feedback to learners, and not forgetting essential information [30].

In addition to lesson-delivery competence, EFL student teachers' public-speaking anxiety was also considered a critical independent factor. Previous research has shown that language anxiety can affect foreign language learners' learning process [31]. Marzec-Stawiarska [15] notes that speaking is often viewed as the most anxiety-provoking aspect of foreign and second language education. Yee and Abidin [32] also highlight that EFL learners who speak in front of peers and teachers may experience heightened nervousness and stress.

Hence, drawing upon prior literature, we have identified five key factors that could impact the self-efficacy (SE) and teaching performance (TP) of student teachers: teaching experience (TE), EFL speaking competence (ESC), lesson-delivery competence (LDC), lesson preparedness (LP), and public-speaking anxiety (PSA).

### 2.2 Research hypotheses

#### 2.2.1 Self-efficacy (SE) and lesson-delivery competence (LDC)

Self-efficacy refers to people's beliefs in their ability to perform certain actions or tasks [[33], [34], [35]]. Teachers' self-efficacy refers to individual teachers' confidence in their ability to plan, organize, and carry out activities that are necessary to achieve educational goals [36,37].

There exists a correlation between self-efficacy and teaching performance. According to Brown, Myers and Collins [37], self-efficacy represents a crucial motivational factor that influences teaching quality and teaching effectiveness. Additionally, lesson-delivery competence can also positively reinforce self-efficacy, as professional training enhances the emotional security and confidence of student teachers [30]. Therefore, we raised the first hypothesis (H1).

H1 Self-efficacy (SE) is positively related to teaching performance (TP).

Lesson-delivery competence is a fundamental aspect of teaching performance that encompasses various verbal and nonverbal skills [38]. Verbal skills include intonation, pronunciation, language fluency, and accuracy, while nonverbal skills comprise body language (e.g., gestures, eye contact, movement), time management, and classroom management [20].

According to Darling-Hammond, Chung and Frelow [39], research and meta-analyses suggest that teachers who receive training to enhance their teaching skills develop higher self-confidence and positive relationships with students [20]. Based on this, we proposed Hypothesis 2.

H2 Lesson-delivery competence (LDC) is positively related to self-efficacy (SE).

#### 2.2.2 Lesson preparedness (LP)

According to Manasia, Ianos and Chicioreanu [20], readiness for teaching is the state of preparedness of student teachers for the teaching profession. Bolliger and Halupa [27] define preparedness as the level of ability or readiness to complete necessary tasks in course delivery. Bolliger and Halupa [27] found a positive relationship between lesson preparedness and teacher self-efficacy. Therefore, we proposed Hypothesis 3.

H3 Lesson preparedness (LP) is positively related to self-efficacy (SE).

#### 2.2.3 Public-speaking anxiety (PSA)

Public-speaking anxiety poses a significant challenge for EFL teachers, hindering their ability to build confidence and persuade students effectively [32]. Previous research show that anxiety experienced by language teachers can decrease their self-confidence (Horwitz, 1996), which, in turn, affects their self-efficacy. Moreover, Merc [40] suggests that foreign language student teachers' anxiety and perceived self-efficacy were negatively correlated. Consequently, we proposed the following hypothesis.

H4 Public speaking anxiety (PSA) is negatively related to self-efficacy (SE).

#### 2.2.4 Teaching experience (TE)

English teaching experience is considered one of the most crucial aspects of EFL teachers' education [41,42]. In terms of lesson-delivery competence, previous studies indicate that teaching experience significantly affects student teachers' tendency towards lesson preparedness [18]. Brown, Myers and Collins [37] suggest that EFL student teachers benefit from their student teaching experiences in terms of preparedness. Additionally, Pasaribu and Harendita [41] support the notion that a lack of teaching experience may result in difficulties and problems, leading to anxiety, which hinders EFL teachers from delivering successful classes. Based on this literature, we proposed the following hypotheses.

H5 Teaching experience (TE) has a positive relationship with lesson-delivery competence (LDC).

H6 Teaching experience (TE) has a positive relationship with lesson preparedness (LP).

H7 Teaching experience (TE) has a negative relationship with public-speaking anxiety (PSA).

#### 2.2.5 EFL speaking competence (ESC)

Speaking competence is a crucial professional skill for teachers as it enables them to apply their professional knowledge in practice (lesson-delivery competence) and reduces lesson preparation time [20,26]. English speaking competence is often considered the primary indicator of an EFL teacher's success [24,25,32].

Daud, Ras, Novitri and Audia [24] suggest that speaking competence is a key contributor to public-speaking anxiety. So, we proposed the following hypotheses.

H8 EFL speaking competence (ESC) is positively related to lesson-delivery competence (LDC).

H9 EFL speaking competence (ESC) is positively related to lesson preparedness (LP).

H10 EFL speaking competence (ESC) is negatively related to public-speaking anxiety (PSA).

Besides, we believe that better lesson preparedness would improve student teachers’ lesson-delivery competence, so the eleventh hypothesis is proposed.

H11 Lesson preparedness (LP) is positively related to lesson-delivery competence (LDC).

**Fig. 1:**
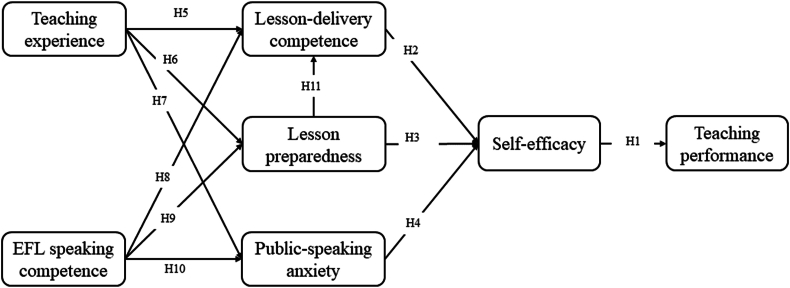
Theoretical framework and hypotheses.

The theoretical framework and hypotheses are depicted in Fig. 1.

## 3 Methodology

This study adopts a quantitative research approach and utilizes convenience sampling to collect survey responses. To ensure data reliability, four validation questions were incorporated to filter out unreliable responses. The study employs various data analysis techniques, such as confirmatory factor analysis, descriptive analysis, correlation analysis, and structural equation modeling (SEM), which are discussed in detail in the Findings section.

### 3.1 Research instruments

To investigate the factors that contribute to EFL teachers' self-efficacy and performance in microteaching training programs, this study employed a survey method to collect data. The survey consists of two demographic items (gender and grade) and seven subscales that measure seven constructs: teaching experience (TE), EFL speaking competence (ESC), lesson-delivery competence (LDC), lesson preparedness (LP), public-speaking anxiety (PSA), self-efficacy (SE), and teaching performance (TP).

1) The TE subscale, which includes seven items such as “I can solve most problems if I invest the necessary effort”, was adapted from scales by Brown, Myers and Collins [37].
2) The ESC subscale, which covers five main aspects (participants' pronunciation, vocabulary, grammar, paraphrase strategies, and general fluency), was developed by the authors with reference to the International English Language Testing System (IELTS) speaking competence assessment criteria.
3) The LDC subscale, containing six items, examines teachers' lesson-delivery competence, such as “I feel I will lose control when introducing new topics to the class” and “I feel I will lose control in catching students' attention”. The LDC subscale was adapted from survey items by Pasaribu and Harendita [41] and Yoon [43].
4) The LP subscale, containing seven items, was adapted from the Measuring Perception of Pre-service Teachers' Preparedness (MPPTP) scale by Brown, Myers and Collins [37].
5) The PSA subscale, which includes six items such as “I feel so nervous that I forget facts I really know while giving a formal microteaching class”, was adapted from the Personal Report of Public Speaking Anxiety (PRPSA) developed by McCroskey [44] and the Foreign Language Student Teacher Anxiety Scale (FLSTAS) by Merc [40].
6) The SE subscale, containing five items, was adapted from the General Self-Efficacy Scale (GSE) by Schwarzer and Jerusalem [45] and the Teacher Sense of Efficacy Scale (TSES) by Tschannen-Moran and Hoy [46].
7) The TP subscale, which includes five items, was adapted from the Subjective Academic Achievement Scale (SAAS) by Stadler, Kemper and Greiff [47].

All survey items, except for the two demographic questions, were 7-point Likert questions ranging from 1 (strongly disagree) to 7 (strongly agree) (see the Appendix).

### 3.2 Reliability and validity

To enhance the reliability and validity of the study, we included one polygraph question, one validation question, and two reverse-scored questions. Table 1 provides details of these items.

**Table 1:** Survey items for better reliability and validity.

Code	Item	Question type
Other 1	“I do not want to get high score in this course.”	Polygraph question
Other 2	Please choose “3-slightly disagree” for this item.	Validation question
PSA 3	“I do not have any fear about giving microteaching classes”	Reversed question
PSA 5	“When giving a microteaching class, I feel totally in control.”	Reversed question

Participants were instructed to disagree with the polygraph question (“I do not want to get a high score in a microteaching course”), select “3” for the validation question (“This question is for validation, please select 3”), and provide opposite answers to the third item measuring EFL student teachers' public speaking anxiety (“I do not have any fear about giving microteaching classes” and “when giving a microteaching class, I feel totally in control”).

Out of the 327 responses, 55 were removed based on these three items, resulting in 272 valid surveys. The valid response rate was 83%.

### 3.3 Participants and ethics considerations

Within an 18-month period, we divided the study into four batches to provide systematic microteaching training to 318 EFL student teachers. Among them, 107 student teachers received 18 h of training (9 weeks), while the remaining participants received 30 h or more of training (15 weeks or more). Afterward, we posted an advertisement in the students' online learning community, which included program information, a consent form, and a survey link. Participants could complete the survey after signing the consent form. We also encouraged participants to share the survey link with peers who had also undergone microteaching training. The entire data collection process spanned one month, and in the end, we gathered responses from 327 EFL student teachers. The entire process was voluntary and anonymous.

After excluding 55 participants (16%) due to insufficient reliability (see Table 2), our final sample size was 272 participants. Of the remaining 272 participants, 39 (14.3%) were male and 233 (85.7%) were female. The majority of participants (n = 140, 51.5%) were postgraduate students (see Table 2).

**Table 2:** Demographic information of the participants.

Demographic variable		Sample	
		Number	Percentage
Gender	Male	39	14.3
	Female	233	85.7
Education level	Bachelor	132	48.5
	Master	140	51.5

Ethical considerations were meticulously addressed throughout the data collection process. Prior to data collection, participants received a comprehensive briefing about the research program. During data collection, participants had the freedom to discontinue their participation at any time. The entire process was conducted anonymously and on a voluntary basis. Approval for this study was granted by the Academic Research Ethics Committee (AREC) of the School of Foreign Languages and Literatures at Chongqing Normal University (approval number: AREC2022SFLL062102).

### 3.4 Data collection and analysis

We administered digital surveys to EFL student teachers using the convenience sampling method. After collecting responses, we removed unreliable data (see Table 2) and imported the remaining data into two software programs, SPSS 23.0 and AMOS 26.0. The specific data analysis processes are detailed in the Findings section.

## 4 Findings

### 4.1 Descriptive data

Table 3 presents the descriptive statistics of seven constructs. The mean scores for the seven variables indicate the average performance levels of the participants in each construct. The highest mean score is for self-efficacy (4.90), indicating that, on average, the participants have high confidence in their ability to perform their teaching duties. The next highest mean scores are for lesson preparedness (4.85), English speaking competence (4.57), and teaching performance (4.57), indicating that the participants are well-prepared, proficient in English, and performing well in their teaching duties, respectively. The mean score for teaching experience is 4.39, indicating that the participants have moderate levels of experience in teaching. The mean score for lesson-delivery competence is 3.66, indicating that this construct has the lowest mean score, suggesting that the participants may have some areas for improvement in their lesson delivery skills. Finally, the mean score for public-speaking anxiety is 3.71, indicating that the participants have moderate levels of anxiety when speaking in front of an audience. Overall, the mean scores suggest that the participants have good to high levels of competence and confidence in their teaching abilities, with some areas for improvement in lesson delivery skills.

**Table 3:** Descriptive statistics.

Construct		Mean	Std. Deviation	Skewness	Kurtosis
Lesson-delivery competence	LDC	3.66	1.376	0.15	−1.05
Lesson preparedness	LP	4.85	1.028	−0.95	0.67
Public-speaking anxiety	PSA	3.71	1.183	0.13	−0.97
Teaching experience	TE	4.39	1.222	−0.30	−0.78
English speaking competence	ESC	4.57	1.205	−0.53	−0.36
Self-efficacy	SE	4.90	1.117	−0.69	0.07
Teaching performance	TP	4.57	1.161	−0.40	−0.33

Skewness measures how asymmetrical a distribution is. A value of 0 indicates perfect symmetry, while positive and negative values indicate right-skewed and left-skewed distributions, respectively. Lesson-delivery competence and public-speaking anxiety have positive skewness values of 0.15 and 0.13, respectively, indicating more scores towards the lower end. The remaining five constructs have negative skewness values ranging from −0.40 to −0.95, indicating more scores towards the higher end. The presence of outliers or extreme values is suggested by the skewness values.

Kurtosis measures distribution peakedness and tails, with 0 indicating a normal distribution and positive/negative values indicating more peaked/flatter distributions. Lesson-delivery competence and public-speaking anxiety have negative kurtosis values (−1.05 and −0.97), indicating flatter distributions, while lesson preparedness has a positive kurtosis value (0.67), indicating a more peaked distribution. The remaining constructs are closer to normal distribution. Though deviations from normality exist, they are not severe enough to invalidate statistical inferences.

### 4.2 Model fit

Several model-fit measures were used to evaluate the goodness of fit of the proposed model, including CMIN/df (Chi-Square divided by degrees of freedom), GFI (Goodness of Fit Index), AGFI (Adjusted Goodness of Fit Index), RMSEA (Root Mean Square Error of Approximation), IFI (Incremental Fit Index), TLI (Tucker-Lewis Index), and CFI (Comparative Fit Index). Fit benchmarks were established according to guidance from Hair et al. (2009), Hu and Bentler (1999), Awang (2015), and Yakubu and Dasuki (2018), and are detailed in Table 4.

**Table 4:** Model fit indices for the measurement model.

Goodness-of-fit measure	x2/df	GFI	AGFI	RMSEA	IFI	TLI	CFI
Recommend value	Between 1 and 3	>0.8	>0.8	≤0.06	>0.9	>0.9	>0.9
Result	1.801	0.801	0.775	0.054	0.929	0.923	0.929

As shown in Table 4, the proposed model demonstrated acceptable overall fit. Specifically, the model exhibited excellent fit with respect to CMIN/df (1.801), RMSEA (0.054), IFI (0.929), TLI (0.923), and CFI (0.929). Although the GFI (0.803) value fell short of the excellent benchmark of 0.9 (Hair et al., 2009), it was higher than the acceptable benchmark of 0.8 (Yakubu & Dasuki, 2018). The AGFI (0.775) was slightly lower than the acceptable benchmark of 0.8. Overall, the proposed model achieved satisfactory model fit.

### 4.3 Internal consistency reliability, convergent validity, and discriminant validity

We employed Cronbach's alpha as a measure of internal consistency reliability, which tests the degree to which different items within a questionnaire or test are measuring the same construct or concept. It is widely accepted that data with a Cronbach's alpha coefficient greater than 0.7 indicates high reliability. A higher Cronbach's alpha coefficient means that the questionnaire will be more stable. The results showed that the internal consistency coefficient of the seven subscales was satisfactory, as they all exceeded the benchmark of 0.7. Specifically, The Cronbach's alpha coefficient value of the overall survey is 0.838, and that for the seven subscales were 0.916 (LDC), 0.896 (LP), 0.804 (PSA), 0.939 (TE), 0.893 (ESC), 0.897 (SE), and 0.907 (TP).

Convergent validity refers to the degree to which different items in a scale that are intended to measure the same construct actually converge or agree with each other in their measurements. To estimate the convergent validity of scale items, we examined three measures of convergent validity: standardized factor loadings (Std. Estimate), composite reliability (CR), and average variance extracted (AVE).

Standardized factor loadings indicate the strength of the relationship between an item and the underlying construct it is intended to measure. We found that most of the scale items had factor loadings above the minimum criterion of 0.70, indicating that they were well-correlated with their intended construct.

Composite reliability measures the extent to which a scale is internally consistent, meaning that the items within the scale are measuring the same construct. We found that all factors had composite reliabilities exceeding the recommended level of 0.70.

Finally, we looked at average variance extracted, which measures the proportion of variance in a construct that is explained by the scale items. We found that all factors except for PSA had average variance extracted values above the threshold of 0.50 (Hair et al., 2006), indicating that the scale items were contributing well to the measurement of the intended construct.

Overall, these results suggest that the scale items were measuring the intended constructs effectively and that the scale as a whole had good convergent validity (see Table 5).

**Table 5:** Convergent validity.

Construct	Item	Unstd. Estimate	S.E.	t-value	P	Std. Estimate	SMC	CR	AVE
LDC	LDC1	1				0.751	0.564	0.917	0.649
	LDC2	1.038	0.079	13.186	***	0.783	0.613		
	LDC3	1.151	0.077	14.856	***	0.872	0.760		
	LDC4	1.010	0.074	13.688	***	0.810	0.656		
	LDC5	0.984	0.075	13.066	***	0.777	0.604		
	LDC6	1.216	0.086	14.193	***	0.836	0.699		
LP	LP1	1				0.726	0.527	0.898	0.558
	LP2	1.052	0.083	12.641	***	0.796	0.634		
	LP3	1.174	0.089	13.238	***	0.835	0.697		
	LP4	1.024	0.085	12.003	***	0.757	0.573		
	LP5	1.081	0.098	11.084	***	0.699	0.489		
	LP6	0.928	0.089	10.443	***	0.660	0.436		
	LP7	1.028	0.088	11.746	***	0.741	0.549		
PSA	PSA1	1				0.879	0.773	0.811	0.439
	PSA2	0.976	0.056	17.378	***	0.894	0.799		
	PSA3	0.66	0.058	11.416	***	0.636	0.404		
	PSA4	0.371	0.06	6.190	***	0.379	0.144		
	PSA5	0.526	0.064	8.181	***	0.485	0.235		
	PSA6	0.551	0.061	9.055	***	0.529	0.28		
TE	TE1	1				0.867	0.752	0.940	0.690
	TE2	0.915	0.045	20.166	***	0.885	0.783		
	TE3	0.799	0.043	18.765	***	0.852	0.726		
	TE4	0.822	0.045	18.458	***	0.845	0.714		
	TE5	0.801	0.050	15.939	***	0.776	0.602		
	TE6	0.752	0.050	15.023	***	0.748	0.56		
	TE7	0.824	0.046	18.041	***	0.834	0.696		
ESC	ESC1	1				0.725	0.526	0.894	0.631
	ESC2	1.392	0.099	14.115	***	0.891	0.794		
	ESC3	1.219	0.089	13.623	***	0.855	0.731		
	ESC4	1.064	0.091	11.757	***	0.738	0.545		
	ESC5	1.054	0.089	11.884	***	0.746	0.557		
SE	SE1	1				0.713	0.508	0.898	0.638
	SE2	1.195	0.090	13.293	***	0.858	0.736		
	SE3	1.231	0.093	13.266	***	0.856	0.733		
	SE4	1.031	0.086	12.03	***	0.772	0.596		
	SE5	1.126	0.092	12.229	***	0.785	0.616		
TP	TP1	1				0.822	0.676	0.907	0.661
	TP2	1.001	0.068	14.737	***	0.793	0.629		
	TP3	1.04	0.07	14.791	***	0.795	0.632		
	TP4	1.044	0.067	15.543	***	0.824	0.679		
	TP5	1.127	0.072	15.715	***	0.831	0.691		

To test for discriminant validity, we employed the Fornell-Larcker criterion approach (Fornell & Larcker, 1971). Firstly, we conducted a confirmatory factor analysis (CFA) to test the measurement model and estimate the factor loadings for each item. Next, we calculated the average variance extracted (AVE) for each construct using the factor loadings of each item. Finally, we compared the square root of the AVE for each construct with the correlation coefficients of that construct with other constructs. Discriminant validity is established when the square root of the AVE is greater than the correlation coefficients between the construct and other constructs.

As indicated in Table 6, most of the correlation coefficients between the constructs are smaller than their corresponding square roots of the AVE. Therefore, the discriminant validity of the current study has been confirmed.

**Table 6:** AVE, the square root of the average variance extracted (AVE) (in bold) and correlations between constructs.

Construct	AVE	Construct						
		LDC	LP	PSA	TE	ESC	SE	TP
LDC	0.649	**0.806**						
LP	0.558	−.528**	**0.747**					
PSA	0.439	.767**	−.531**	**0.663**				
TE	0.690	−.673**	.720**	−.621**	**0.831**			
ESC	0.631	−.653**	.706**	−.620**	.747**	**0.794**		
SE	0.638	−.611**	.765**	−.582**	.727**	.778**	**0.799**	
TP	0.661	−.637**	.779**	−.599**	.789**	.783**	.840**	**0.813**

### 4.4 Path analysis

**Fig. 2:**
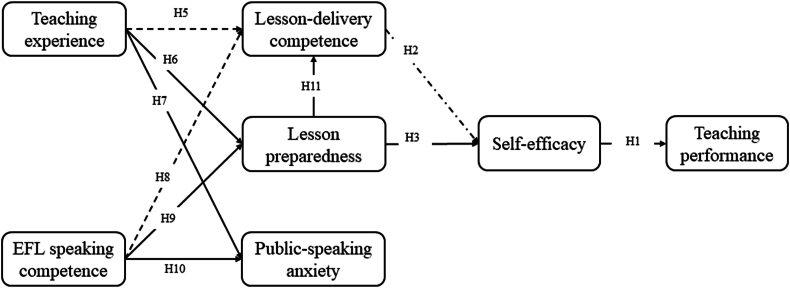
The validated framework (Note: the dashed line represents a negative relationship).

Table 7 and Fig. 2 depict path coefficients of the proposed research model, which revealed that seven out of 11 hypotheses were supported by the data.

**Table 7:** Hypotheses testing result.

#	Path	Hypothesis	Result	Estimate	P-value	Hypothesis-testing result
H1	SE-TP	Positive	Positive	0.886	***	Supported
H2	LDC-SE	Positive	Negative	−0.224	***	Unsupported
H3	LP-SE	Positive	Positive	0.939	***	Supported
H4	PSA-SE	Negative	Negative	−0.072	0.205	Unsupported
H5	TE-LDC	Positive	Negative	−0.557	***	Unsupported
H6	TE-LP	Positive	Positive	0.308	***	Supported
H7	TE-PSA	Negative	Negative	−0.29	***	Supported
H8	ESC-LDC	Positive	Negative	−0.651	***	Unsupported
H9	ESC-LP	Positive	Positive	0.404	***	Supported
H10	ESC-PSA	Negative	Negative	−0.289	***	Supported
H11	LP-LDC	Positive	Positive	0.412	**	Supported

Specifically, the results demonstrate that lesson preparedness (LP) significantly influences self-efficacy (SE) (*β* = 0.939, *p* < 0.001), and self-efficacy (SE) significantly impacts student teachers' teaching performances (TP) (*β* = 0.086, *p* < 0.001). Therefore, hypotheses H3 and H1 are supported.

We hypothesized that students who are more competent in delivering lessons would experience higher self-efficacy. However, contrary to expectations, the data revealed a negative relationship between lesson-delivery competence (LDC) and self-efficacy (SE) (*β* = −0.224, *p* < 0.001). Additionally, it was anticipated that public-speaking anxiety (PSA) would negatively affect student teachers' self-efficacy. However, the results showed that the two variables are not statistically or significantly related (*p* = 0.205). As a result, hypotheses H2 and H4 are not supported.

It is predicted that teaching experience, as a critical factor in the development of teaching ability, has a positive influence on lesson-delivery competence (H5) and lesson preparedness (H6), and a negative relationship with public-speaking anxiety (H7). While H6 and H7 were supported, H5 was not. The data suggest that teaching experience has a negative impact on student teachers’ lesson-delivery competence (LDC) (*β* = −0.557, *p* < 0.001). In other words, the participants reported that the more teaching experience they had, the less effective they were at delivering lessons.

EFL speaking competence (ESC) refers to an EFL learner's ability to effectively communicate in spoken English. For EFL student teachers, mastering another language could be as challenging as mastering teaching skills. The hypotheses were that student teachers with higher ESC would be better prepared in lesson planning (H9) and experience less anxiety when giving public speeches during microteaching training programs (H10). H9 and H10 were confirmed. However, H8 was unsupported since the β value is negative (*β* = −0.651, *p* < 0.001), despite the hypothesis that EFL speaking competence should positively relate to lesson-delivery competence.

H11 indicates that lesson preparedness (LP) is positively related with lesson-delivery competence (LDC), because teachers who are thoroughly prepared for a lesson are more likely to have a comprehensive understanding of the material, which enhances their capability to effectively communicate the lesson to their students. Moreover, a well-prepared teacher is typically more self-assured, structured, and adaptable to unforeseen circumstances during the lesson, all of which are crucial factors that facilitate successful lesson delivery. Results show that there is a positive relationship between LP and LDC (*β* = 0.412), and the relationship is statistically significant (*p* = 0.008, <0.01).

To sum up, we confirmed seven hypotheses from 11. Among the four unconfirmed hypotheses, one lacked statistical significance (H4), while the other three had a reversed positive-negative relationship. These three hypotheses suggested that teaching experience, EFL speaking competence, and lesson-delivery competence, as well as self-efficacy, were positively related, but none of them were supported by the data.

## 5 Discussion

The results show that the respondents think their teaching ability is quite weak: on a 7-point Likert scale, their self-rated score for teaching ability was only 3.66. “3” means “slightly disagree”, “4” means “don't know” or “neutral”, and “5” means “slightly agree”. An average score of 3.66 indicates that respondents are generally dissatisfied with their teaching ability. The variance for this item is also the highest (SD = 1.376), meaning that respondents perceive a large difference in their teaching ability. However, students' self-efficacy is high, ranking first in all dimensions (Mean = 4.90, SD = 1.117).

H2, H4, H5, and H8 were not confirmed. The underlying reasons need further exploration. The possible explanations for these findings are detailed below.

### 5.1 Why lesson-delivery competence is negatively and significantly related with self-efficacy?

The unsupported H2 indicates that lesson-delivery competence is negatively and significantly related with self-efficacy, namely the more capable one feels in lesson-delivery, the lower self-efficacy he or she will perceive. This result is inconsistent with the previous hypothesis [20,39].

We propose that the theory of knowledge illusion, also known as the illusion of explanatory depth, can account for the aforementioned phenomenon. This theory suggests that people tend to overestimate their level of understanding or knowledge of a particular subject, topic, or system [48]. For instance, individuals with limited experience or knowledge of a subject may underestimate its complexity and believe they know very little, while those with some knowledge may overestimate their understanding and feel like they know a lot [48]. It is only as individuals deepen their knowledge that they begin to realize the gaps in their understanding. In the context of microteaching, student teachers with limited training may be overly confident due to underestimating the complexity of the task, while those who understand its complexity may find the training more challenging. Therefore, student teachers who have better lesson-delivery competence can have slower self-efficacy.

Similarly, we believe that student teachers have varied expectations. People who are confident in their ability to deliver lessons may set high expectations for themselves, which can result in lower self-efficacy if they do not meet these expectations. On the other hand, individuals who are highly skilled in lesson-delivery may be more self-critical, which can also lead to lower self-efficacy [36,37]. However, higher goal-setting boosts motivation, which in turn improves self-efficacy or performance [49]. Therefore, we suggest future studies to interpret the findings with caution.

### 5.2 Why public-speaking anxiety is not significantly related with self-efficacy?

The unsupported H4 implies that while there is a negative correlation between students' public speaking anxiety and self-efficacy, this correlation is not statistically significant. We propose that there are four potential explanations for this result: a shortage of scales that measure moderate levels of anxiety in learning contexts, a failure to consider anxiety facilitation, an assumption regarding the severity of public speaking anxiety, and biases in data collection.

Firstly, there is a lack of scales measuring moderate level of anxiety in learning contexts. Anxiety has primarily been studied in medical care fields, leading to an emphasis on somatic anxiety, which is typically seen as the most debilitating form of anxiety [31]. Somatic anxiety, also known as somatization, can manifest as physical symptoms like chest pain, headache, dyspepsia, insomnia, and dizziness [31]. However, anxiety in learning contexts can take on a different form. Anxious learners may be reluctant to complete learning tasks, but they are less likely to experience severe somatic symptoms like stomachaches and insomnia [31]. We were aware of this issue when selecting research instruments; however, the lack of appropriate scales still had an impact on the results.

Secondly, anxiety can be categorized into two types: debilitating anxiety and facilitating anxiety, but the latter one is often neglected. According to Luo, Subramaniam and O'Steen [31], anxiety has a positive and significant correlation with positive emotions, such as motivation, due to the presence of facilitating anxiety. Facilitating anxiety is defined as the anxiety that contributes to good performances [50].

Strack and Esteves [51] and Strack, Lopes, Esteves and Fernandez-Berrocal [52] extensively explain this phenomenon by stating that anxiety serves as an indicator of potential problems or threats, making individuals more vigilant to avoid unforeseen circumstances. Consequently, when individuals perceive something as important, they tend to feel anxious, leading them to invest more time and effort in achieving their goals. Ultimately, this facilitative anxiety promotes persistence and high levels of motivation, which are associated with improved performance and outcomes. Failing to consider facilitating anxiety results in an incomplete understanding of anxiety, as anxiety is often seen solely as a negative factor that leads to poor performance [31]. The present study does not differentiate between facilitating anxiety and debilitating anxiety, which may reduce the precision of the results.

Thirdly, public-speaking anxiety may not be a severe problem in microteaching training programs. Although delivering speeches can be anxiety-provoking, student teachers in microteaching training programs give speeches to their classmates, who they are already familiar with. Accordingly, the student teachers’ anxiety level can be considerably lower. Additionally, microteaching training provides a safe and non-threatening environment for student teachers, enabling them to make mistakes, reflect on the mistakes, and correct the mistakes [2,8,9]. At the same time, we believe that the student teachers may hold the belief that “I am still a student, and making mistakes is a natural part of the learning process”. By adopting this mindset, the act of giving public speeches becomes significantly more comforting.

Moreover, even though student teachers may initially feel anxious, their anxiety tends to decrease over time with training. When collecting data, we found that participants had already undergone microteaching training for more than two months, indicating that their public-speaking anxiety levels are not as high as we initially assumed.

Fourthly, biases may be present due to the method of data collection [53,54]. Specifically, two out of the six items used to measure public-speaking anxiety are phrased in a reverse manner, which could lead to confusion or inaccurate responses from participants. Additionally, the possibility exists that participants may have completed the form hastily, without carefully considering each item, which could negatively impact the accuracy of the research findings [55].

### 5.3 Why teaching experience is negatively related with lesson-delivery competence?

The untested H5 suggests that teaching experience is negatively and significantly related to lesson-delivery competence. We believe this might be due to the possibility that participants' reported teaching experience differs from the commonly assumed actual school immersion. Moreover, as participants gain more experience, they may become more aware of their knowledge gaps, which can subsequently influence their performance in Microteaching training.

Firstly, the teaching experience reported by the participants may differ from what is commonly assumed as actual school immersion. Some participants indicated that their experience came from voluntary teaching activities, such as teaching English songs in summer camps or providing introductions to English-speaking countries to high school students. These teaching activities primarily emphasize enjoyable learning experiences rather than specific pedagogical objectives. This is in stark contrast to the requirements of microteaching, which necessitate adherence to clear instructional design principles, the achievement of well-defined educational goals, the promotion of teaching implementation following established protocols, and the demonstration of specific teaching skills at precise stages [7]. Consequently, even though participants perceive themselves as having experience, they may still reveal shortcomings in their lesson-delivery competence during microteaching training.

Similarly, student teachers are often not assigned to teach large classes due to their qualifications. Even when they do get teaching opportunities, it is usually in small groups as teaching assistants rather than in the role of full-fledged teachers. However, microteaching training is to prepare teachers for teaching in large classes, which is why this study involves the dimension of public speaking anxiety [2]. These differences mean that participants who self-report teaching experience may not actually possess a significant advantage.

Moreover, we propose that EFL student teachers who have relevant experience may have a clearer awareness of their lack of pertinent knowledge. The more exposure they have to real teaching situations, the more they come to realize their deficiencies in various areas, including subject matter content knowledge, pedagogical content knowledge, and curricular knowledge [30]. These deficiencies can directly impact their lesson delivery performance, encompassing aspects such as the selection of teaching strategies, classroom management, and communication skills with students. Therefore, even if they have relevant teaching experience, it does not necessarily indicate that student teachers will perform better in microteaching training [30].

It's worth noting that the aforementioned points are solely our assumptions, as they are based on informal discussions with students rather than formal data collection.

### 5.4 Why EFL speaking competence is negatively and significantly related with lesson-delivery competence?

It is assumed that student teachers who are not confident in their own EFL-speaking abilities may be less confident and less effective in delivering EFL lessons [30,36,37]. However, the unsupported H8 indicates that EFL speaking competence is negatively and significantly related with lesson-delivery competence.

The findings contradict previous research and are counterintuitive. The only possible explanation we can think of is that we adapted the IELTS speaking criteria to assess participants' EFL speaking competence. Although the items demonstrated good internal consistency reliability, they were not proved suitable for the current study. Future studies could consider using alternative measurement criteria or scales, or conducting a duplicate study with a different group of participants.

## 6 Conclusion

### 6.1 Summary

In conclusion, this study has developed a theoretical framework that illuminates the factors influencing the self-efficacy and teaching performance of EFL student teachers in microteaching trainings, encompassing TP, SE, LDC, LP, and PSA. We tested eleven hypotheses, with seven finding support in the data, while four remained unconfirmed.

The unsupported H2 suggests a negative and significant correlation between lesson-delivery competence and self-efficacy, a phenomenon explained by the theory of knowledge illusion and varying learner expectations.

The unsupported H4 implies a negative correlation between students' self-efficacy and public speaking anxiety, which can be attributed to several factors, including the absence of scales measuring moderate anxiety levels in learning contexts, a lack of consideration for anxiety facilitation, assumptions about the severity of public speaking anxiety, and biases in data collection.

The unsupported H5 indicates a negative and significant relationship between teaching experience and lesson-delivery competence, primarily because participants' experiences do not align with the requirements of microteaching training programs.

The unsupported H8 suggests a negative and significant relationship between EFL speaking competence and lesson-delivery competence, warranting further investigation.

These research findings offer valuable insights for teacher education. Teacher educators should pay attention to the confidence of student teachers who excel in delivering classes, as these individuals may grapple with self-efficacy issues due to their own high expectations and self-criticism. Moreover, when assessing anxiety levels in teacher training programs, educators should consider measuring facilitating anxiety in learning contexts and providing corresponding guidance [56].

### 6.2 Limitations and future research

While this study has yielded valuable insights, it is not exempt from limitations. Firstly, this research primarily adopts a quantitative approach, which limits the depth of interpretations when explaining the four hypotheses that were rejected. While we supplemented these findings with participant interactions, these explanations lack the rigor of data obtained through comprehensive data collection. As suggested by Luo (2022), future studies could adopt a mixed sequential approach, combining both qualitative and quantitative research. Employing the explanatory mixed sequential method, researchers can begin with a quantitative analysis to explore the relationships between variables and subsequently conduct a qualitative study to delve deeper into the underlying reasons behind these relationships or findings.

Furthermore, in this study, the assessment of EFL speaking competence relied on the IELTS scoring criteria. While this criterion is theoretically sound and logically acceptable, it yielded an unexpected result: a negative and significant relationship between EFL speaking competence and lesson-delivery competence. Future research should further investigate this result and provide comprehensive explanations.

Moreover, it's important to note that the data collected in this study is specific to one university, and caution should be exercised when extrapolating the findings to other regions. Future studies could enhance the generalizability of these findings by introducing additional variables, expanding the sample size, and recruiting participants from various countries and cultural backgrounds.

Additionally, in the era of increasing online teaching, future research should also explore teachers' preparedness for online instruction.

## Ethics statement

All procedures performed in studies involving human participants were in accordance with ethical standards. Informed consent was obtained from all individual participants included in the study. The study was approved by the Academic Research Ethics Committee of the School of Foreign Languages and Literature in Chongqing Normal University (approval number: AREC2022SFLL062102).

## Funding information

This study was supported by the Humanities and Social Sciences 10.13039/100003536Youth Foundation, 10.13039/501100002338Ministry of Education of the People's Republic of China (grant number: 23YJC880074) and 10.13039/100010338Chongqing Normal University (grant number: 202334).

## Data availability statement

Data of the current study is available on Figshare. Link: https://doi.org/10.6084/m9.figshare.22548745.v1.

## CRediT authorship contribution statement

**Zhanni Luo:** Writing – review & editing, Writing – original draft, Validation, Supervision, Resources, Methodology, Funding acquisition, Formal analysis, Conceptualization. **Huazhen Li:** Writing – original draft, Investigation, Data curation, Conceptualization.

## Declaration of competing interest

The authors declare that they have no known competing financial interests or personal relationships that could have appeared to influence the work reported in this paper.

## Appendix

Survey items measuring teaching experience (TE).

1. I describe myself as an experienced teacher in teaching the lead-in section.
2. I feel I am experienced in the presentation section.
2. I feel I am experience in the practice section.
4 I feel I am experience in the production section.
5 I feel I am experience in the assignments or review section.
6 I feel I am experience in the making teaching (materials) PPT slides and blackboard design.
7 Generally, I feel I am experience to teach the class.

Survey items measuring EFL speaking competence (ESC).

1. My pronunciation is sufficient for my microteaching delivery.
2. My vocabulary is sufficient for my microteaching delivery.
3. My grammar knowledge is sufficient for my microteaching delivery.
4. I can use strategies to describe difficult words (e. g. use “the thing we use to drink water” to replace “bottle”) when delivering a microteaching class.
5. My English fluency is sufficient for my microteaching delivery.

Survey items measuring lesson-delivery competence (LDC).

1. I feel I will lose control when introducing new topics to the class.
2. I feel I will lose control when delivering abstract concept to the class.
3. I feel I will lose control in catching students' attention.
4. I feel I will lose control in reaching the teaching goals.
5. I feel I will lose control in giving appropriate feedback to listeners.
6. When delivering a microteaching, I get so nervous that I forget facts I really know.

Survey items measuring lesson preparedness (LP).

1. I feel prepared for teaching the lead-in section.
2. I feel prepared for teaching the presentation section.
3. I feel prepared for leading the practice activities.
4. I feel prepared for leading the production activities.
5. I feel prepared for assigning homework or leading students to review.
6. I am ready for my material preparation (e. g., PPT slides).
7. Generally, I feel prepared for my microteaching practice.

Survey items measuring public-speaking anxiety (PSA).

1. I feel anxious when preparing for a formal microteaching class.
2. I feel anxious when I think about a formal microteaching class coming up.
3. (Reverse question) I feel no fear to give a formal microteaching class.
4. I feel that I have trouble falling asleep the night before a formal microteaching.
5. l feel that l am in complete possession of myself while giving a formal microteaching class.
6. I feel so nervous that I forget facts I really know while giving a formal microteaching class.

Survey items measuring self-efficacy (SE).

1. I can always manage to solve difficult teaching problems if I try hard enough.
2. I can stick to my teaching aims and accomplish my goals.
3. I can remain calm when facing teaching difficulties because I can rely on my teaching abilities.
4. I can solve most problems if I invest the necessary effort.
5. I can usually handle whatever comes my way.

Survey items measuring teaching performance (TP).

1. I am satisfied with my knowledge presentation.
2. I am satisfied with my practice activities.
3. I am satisfied with my production activities.
4. I am satisfied with my teaching competence.
5. I can get high score in teaching examination.
